# Multimorbidity among indigenous tribal communities in Kerala, India: a cross-sectional study (2022–2024)

**DOI:** 10.1016/j.lansea.2026.100781

**Published:** 2026-05-14

**Authors:** Panniyammakal Jeemon, Sunaib Ismail, Mathew J. Valamparampil, Jissa Vinoda Thulaseedharan, Srinivas Gopala

**Affiliations:** Sree Chitra Tirunal Institute for Medical Sciences and Technology, Trivandrum, India

**Keywords:** Multimorbidity, Health inequities, India, Noncommunicable diseases, Tribal populations, Cross-sectional study

## Abstract

**Background:**

The burden of multimorbidity is increasing in India, particularly in the state of Kerala. Despite advancement in primary care, several inequities persist among underserved tribal communities. We assessed the prevalence and determinants of non-communicable disease multimorbidity among the indigenous adult (≥30 years) population belonging to tribal communities in Kerala.

**Methods:**

We conducted a community-based cross-sectional survey among 18 Indigenous tribal communities in Kerala from September 2022 to July 2024. Adults who were older than or equal to 30 years and residing in selected hamlets were included in the study. We collected sociodemographic, behavioural, clinical, and anthropometric data using standard tools. Further, we measured blood pressure, point-of-care glucose, creatinine, haemoglobin, and urine biomarkers (sodium, potassium, creatinine, albumin). Detailed medical records and clinical histories were systematically reviewed to ascertain the prevalence of non-communicable diseases (NCDs). Data analysis was conducted using descriptive statistics, multivariable regression, and disease co-occurrence network analysis (Louvain clustering) in R (version 4.4.3).

**Findings:**

A total of 2333 adults (≥30 years) belonging to tribal communities were included in the study. The mean age (±SD) of the participants was 50.0 ± 13.2 years and three-fifths (n = 1356) of them were women. Cardio-metabolic conditions, including hypertension (50.8%), diabetes (32.2%), and chronic kidney disease (22.6%), were the most common NCDs. The overall prevalence of multimorbidity was 55.2% (95% CI 52.7–57.6). The prevalence was 39.5% among adults 30–49 years and 87.3% among those older than 70 years of age. Older age group had higher odds of multimorbidity, with adjusted odds ratios (AORs) ranging from 2.4 to 8.0. Low BMI (AOR 1.8, 95% CI 1.2–2.8) and abdominal obesity (AOR 2.1, 95% CI 1.4–3.0), were also associated with multimorbidity. Network analysis revealed a dominant cardio-metabolic cluster linking hypertension, diabetes, chronic kidney disease, and anaemia.

**Interpretation:**

Multimorbidity is prevalent in one out of two adults (≥30 years) among indigenous tribal communities in Kerala. Cardiometabolic and renal diseases often co-occur. The findings underscore the need for integrated primary care, targeted screening, and culturally tailored interventions to address chronic disease burden in Indigenous population.

**Funding:**

This project is funded by the Department of Science and Technology (DST), Government of India (Project 6117).

**Research in context**

**Evidence before this study**

We conducted a PubMed search for studies published up to 13 April 2026, employing relevant terms such as multimorbidity, comorbidity, chronic disease, prevalence, epidemiology, India, low- and middle-income countries, disease clustering, and network analysis, along with appropriate MeSH terms. This yielded 444 initial records, from which 22 studies were deemed relevant, following screening for those analysing patterns or clustering of co-occurring diseases. Prior research has documented an increasing multimorbidity burden with advancing age and highlighted common cardiometabolic disease clusters. Yet, evidence from India largely stems from cross-sectional surveys relying on condition counts or pairwise correlations. Mostly studies are based on self-reported conditions with scant investigation into intricate disease interrelationships. Moreover, community-based data on multimorbidity patterns across sociodemographic groups, particularly via network-based methods, remain relatively scarce, alongside limited representation of vulnerable indigenous populations.

**Added value of this study**

Our study on multimorbidity among adults older than or equal to 30 in 18 indigenous tribal populations in Kerala shows that more than half of the participants were affected. We also observed a rise in prevalence of multimorbidity with advancing age, a strong clustering of cardio-metabolic conditions and both low BMI and abdominal obesity were independently associated with multimorbidity. Our study contributes to the existing evidence by examining multimorbidity as a system of interacting conditions rather than as simple counts or pairwise associations. By applying network analysis, this study helps to address gaps in understanding how multimorbidity patterns manifest in vulnerable indigenous populations in India.

**Implications of all the available evidence**

Taken together with existing evidence, our findings support a shift from single-disease approaches towards integrated strategies for the prevention and management of chronic conditions. The identification of consistent cardiometabolic clusters underscores the importance of targeting shared risk factors through coordinated screening and management programmes. Future research should prioritise longitudinal and intervention studies to better understand the evolution of multimorbidity and to evaluate strategies that address clusters of conditions rather than individual diseases in isolation.

## Introduction

Multimorbidity, defined as the coexistence of two or more non-communicable diseases (NCDs), is an important public health challenge worldwide.^1^ Global evidence indicates that multimorbidity disproportionately affects older adults and socially vulnerable populations, contributing to increased healthcare utilisation, reduced quality of life, and premature mortality.^1^^,^^2^ In India, the prevalence of multimorbidity among adults varies widely,^3^ affecting almost 50% of older populations and the burden is particularly high in southern states such as Kerala.^4^

Indigenous tribal population, who constitute about 8.6% (104 million) of India’s total population,^4^ have traditionally been viewed as vulnerable to health disparities due to social marginalisation, distinct dietary and lifestyle practices, and limited access to quality health care.^5^ The tribal communities in India face persistent inequities in health, education, and access to care, reflected in their lower life expectancy (∼60 years) compared with the national average of 69 years.^5^ Recent studies show an emerging trend where there is a shift in pattern of disease with multimorbidity becoming increasingly evident among India’s tribal communities.^6^ The rise in burden of NCDs, such as hypertension and diabetes, among tribal populations is often accompanied by persistent issues like infectious diseases and undernutrition.^7^ This intersection of chronic and infectious diseases pose unique challenges for health system and underscores the need for context-specific interventions.^8^ Socioeconomic challenges, lower health literacy, and geographical barriers compound these intersecting burdens. In Kerala, tribal populations are primarily concentrated in remote and underserved regions. Despite advancement in primary care infrastructure of the state, tribal communities continue to face health inequalities related to chronic NCDs.

Preliminary data from hospital- and community-based studies highlights that age, gender, behavioural factors like tobacco and alcohol use, and social exclusion influence multimorbidity among tribal population.^9^ However, there is limited evidence on NCD multimorbidity among vulnerable tribal populations, particularly regarding patterns of co-occurring conditions, which is essential for the development of effective prevention and management strategies.^10^ Although prior studies in India have described the prevalence of multimorbidity using counts or regression-based approaches, few have examined patterns of co-occurrence in tribal populations.^10^^,^^11^ The objectives of this study were to estimate the prevalence of multimorbidity among adults belonging to tribal communities in Kerala, examine patterns of co-occurring chronic conditions using a network-based approach, and to assess associated sociodemographic and behavioural factors.

## Methods

A community-based, cross-sectional study was conducted among adults older than or equal to 30 years in tribal settlements across three districts of Kerala (Malappuram, Wayanad, and Thiruvananthapuram) between September 2022 and July 2024. The age cut-off 30 years was selected in accordance with the Government of India’s National Programme for Prevention & Control of Non-Communicable Diseases (NP-NCD), which recommends population screening from 30 years onward due to the substantially increased risk of lifestyle-related chronic conditions such as hypertension and diabetes.^12^ Districts were selected to represent diverse tribal communities and geographic contexts in Kerala. The population size of the tribal community in the selected districts was 2,01,192. Using stratified random sampling, 25 hamlets per district were selected, and all eligible adults in the selected hamlets were invited to participate. A comprehensive listing of tribal hamlets in each of the three study districts was obtained from official records and verified with local authorities. The hamlets were then stratified by population size to ensure adequate representation of both small and large settlements. From each district, 25 hamlets were selected using simple random sampling. All eligible adults older than or equal to 30 years residing in the hamlets chosen were invited to participate in the study.

**Fig. 1:**
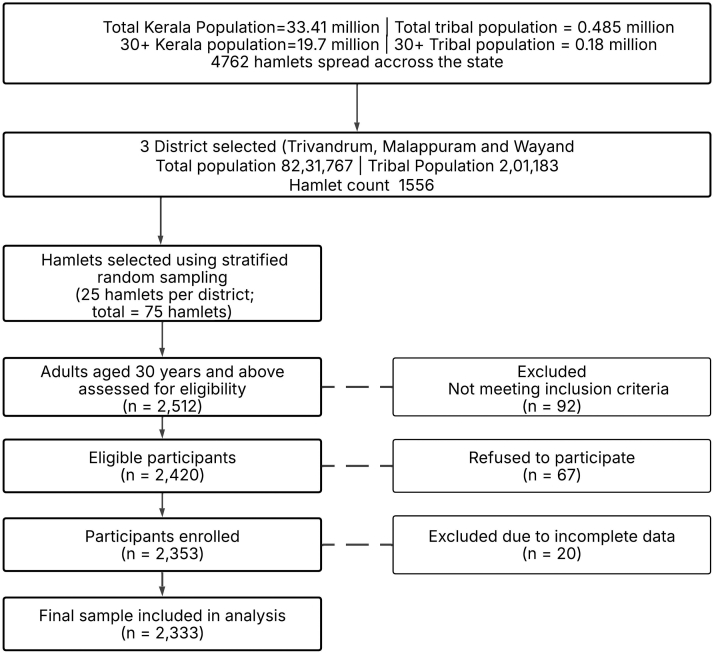
Participant recruitment flowchart.

The sample size for the study was estimated considering an expected prevalence in multimorbidity of 23% (based on data from LASI wave 1: 2017–18),^13^ with a relative precision of 10% (corresponding to an absolute precision of 2.3%) and a 95% confidence level. To account for the potential effect of clustering at the hamlet level, a design effect of 1.5 was incorporated into the calculation, resulting in a minimum required sample size of 1924 participants. To account for potential non-response or incomplete data, the sample size was increased by 15%, resulting in a final target sample of 2264 participants. However, as all eligible individuals were included, a total of 2333 participants were enrolled and included in the final analysis (Fig. 1).

A trained research team comprising a study coordinator and qualified research nurses with prior experience in community-based health surveys was engaged for data collection at each site. The team underwent orientation and field training sessions to ensure uniformity in data collection procedures and adherence to ethical standards. Before commencing the survey, a series of consultative meetings was organised with tribal leaders, local health workers, and representatives from the *panchayat* (a local self-government institution) in each study district. These meetings were designed to foster trust with the community, clarify the study’s purpose, objectives, and potential benefits, and address any concerns related to participation. The involvement of local authorities and community representatives helped facilitate smooth coordination during fieldwork and enhanced participant engagement.

### Procedures

We used a structured questionnaire to collect data on participants’ demographic characteristics, education, and lifestyle risk factors, including alcohol and tobacco consumption.

The field team assessed tobacco use with the Fagerström Test for Nicotine Dependence,^14^ (for both smoking and smokeless forms), alcohol consumption with the Alcohol Use Disorders Identification Test (AUDIT),^15^ physical activity using the Global Physical Activity Questionnaire (GPAQ),^16^ and dietary intake through a 24-h dietary recall. The research nurses in the field team evaluated mental health using the Patient Health Questionnaire-9 (PHQ-9)^17^ for depressive symptoms and the Generalised Anxiety Disorder-7 (GAD-7)^18^ for anxiety. They measured health-related quality of life using the EQ-5D-5L instrument and assessed treatment burden with the Multimorbidity Treatment Burden Questionnaire (MTBQ).^19^

The research nurses in the study recorded anthropometric measurements following standardised procedures. They measured height to the nearest 0.1 cm using a stadiometer and weight to the nearest 0.1 kg with a calibrated digital scale, both of which were placed on level ground. They also measured waist circumference midway between the lower rib margin and the iliac crest with a non-stretchable measuring tape. The research nurses measured blood pressure (both systolic and diastolic blood pressure) in the non-dominant arm. The subject was seated, and the measurement was taken using an automated digital sphygmomanometer after a rest period of at least 5 min. They took three readings at 1-min intervals. The average of three blood pressure values was used in the analysis. The research nurses assessed handgrip strength as an indicator of muscle function and cardiovascular risk using a calibrated hand dynamometer. The participants were seated on a straight-backed chair with their elbow flexed at a 90-degree angle and instructed to squeeze the device with maximum effort using their dominant hand. The research nurses recorded three readings at 15-s intervals and calculated the average for analysis. The participants with recent hand injuries, pain, or surgery were excluded from this test. Comprehensive information on instrument brands, models, and calibration protocols is available in the Supplementary file (Supplementary Table S2). Information on long-term conditions was collected through structured, interviewer-administered questionnaires.

Most conditions, including heart disease, respiratory disease, arthritis, epilepsy, cancer, sickle cell anaemia, and mental disorders, were self-reported, with unclear responses clarified by the research nurses using locally understandable terms, medication history, and prior clinical advice. Only conditions previously diagnosed by a healthcare provider were recorded to minimise misclassification. For hypertension, diabetes, and depression, active measurements (blood pressure, fasting glucose, and PHQ-9) were used and supplemented by self-reported diagnoses. The research nurses collected spot urine samples to estimate sodium, potassium, creatinine, and the albumin-to-creatinine ratio. They also obtained capillary blood samples from the index finger using a sterile, single-use lancet to measure fasting blood glucose, HbA1c, haemoglobin, serum creatinine, and lipid profile with validated point-of-care devices. The study team informed participants in advance about the requirement for an overnight fast. They made up to three attempts, including two revisits, to collect fasting samples; participants who could not provide fasting samples were excluded from biochemical analyses. The field team segregated biomedical waste generated during field activities and ensured its safe disposal through authorised local health facilities in accordance with biomedical waste management protocols. Details of urine analyses performed in a NABL-accredited laboratory, along with the certified point-of-care devices used for haematologic measurements, are provided in the Supplementary file (Online supplement, Supplementary Table S2).

Multimorbidity was defined as the co-occurrence of two or more chronic non-communicable disease conditions within an individual.^20^ Each condition was operationalised using a combination of self-reports, prior clinical diagnoses, and objective measurements where available. The chronic conditions included in the multimorbidity analysis were hypertension, diabetes mellitus, chronic kidney disease, anaemia, heart disease, chronic respiratory disease, arthritis, epilepsy, cancer, sickle cell anaemia, depression, anxiety, and other mental disorders (Online supplement, Supplementary Table S3). Each condition was equally weighted, with the presence of any condition contributing one point toward the multimorbidity count. These were selected a priori based on their relevance to the chronic disease burden in the study population and the availability of reliable survey data. Hypertension was identified according to the Joint National Committee (JNC-7) guidelines^21^ or based on a self-reported history of treatment for hypertension. Diabetes mellitus was defined as a fasting blood sugar (FBS) level ≥ 126 mg/dL^22^ or a self-reported history of diabetes treatment. Chronic respiratory disease was defined as self-reported asthma or COPD or identified based on the COPD-PS screening score. Depression was defined as a score of 10 or higher on the Patient Health Questionnaire-9 (PHQ-9),^17^ and anxiety was defined as a score of 10 or higher on the Generalized Anxiety Disorder-7 (GAD-7).^18^ Chronic kidney disease (CKD) was classified according to the Kidney Disease: Improving Global Outcomes (KDIGO) guidelines and corresponding risk stratification.^23^ Anaemia was defined using World Health Organisation (WHO) criteria as a haemoglobin concentration below 13.0 g/dL in men and above 12.0 g/dL in women.^24^ Abdominal obesity was defined using waist circumference thresholds for South Asians: >90 cm for men and >80 cm for women.^25^ This manuscript was developed in accordance with the STROBE (Strengthening the Reporting of Observational Studies in Epidemiology) guidelines.^26^ We used a STROBE checklist to ensure the completeness and transparency of our reporting (Online Supplement, Supplementary Table S1).

### Statistical analysis

We conducted descriptive analyses to summarise the socio-demographic, behavioural, and clinical characteristics of the study population. The prevalence of multimorbidity was estimated with 95% confidence intervals with standard errors adjusted using a design effect of 1.5 to account for clustering at the hamlet level. Specifically, we multiplied the lower and upper limits of the 95% CI by the square root of the design effect. We presented continuous variables as mean and standard deviation and categorical variables as frequencies and percentages. Age was categorised into four groups (30–49, 50–59, 60–69, and ≥70 years) for regression analyses, while a simplified categorisation (≤50 and >50 years) was used for descriptive presentation of participant characteristics. To ensure valid comparisons across sex, age groups, and other subgroups, we applied age-standardized prevalence estimates.

To characterise patterns of co-occurrence between chronic conditions, we applied network analysis, conceptualising multimorbidity as a system of interacting diseases (Online supplement, Supplementary Table S4). We performed network analysis on pooled individual-level data. Hamlet-level clustering was not explicitly adjusted for, due to the exploratory nature of the network approach. Unlike conventional approaches based on condition counts or pairwise associations, this approach examined relationships between multiple conditions simultaneously. We constructed a weighted, undirected disease co-occurrence network in which nodes represented individual chronic conditions and edges represented pairwise co-occurrence. Edge weights were defined as the joint prevalence (i.e., the percentage of participants with both conditions) derived from binary disease indicators. In the primary analysis, all disease pairs with non-zero co-occurrence were retained. Community structure was identified using the Louvain algorithm. All chronic conditions were included in the network; conditions with very low prevalence, less than 5% (cancer, mental health illness, epilepsy, and sickle cell anaemia) were excluded from clustering analyses. Disease clusters were identified using the Louvain community detection algorithm, which partitions the network into communities by maximizing modularity. The Louvain method is computationally efficient and widely used for detecting community structure in weighted networks.^27^ The algorithm was applied to the weighted undirected co-occurrence network using the implementation in the igraph package in R with default parameters. Communities detected by the algorithm were interpreted as clusters of diseases that co-occur more frequently within the network. Modularity was calculated to quantify the strength of community structure. As a sensitivity analysis, we constructed networks using an alternative edge definition based on the co-occurrence thresholds and Jaccard index to assess the stability of identified clusters. Multivariable logistic regression models were used to examine sociodemographic and behavioural factors associated with multimorbidity. Covariates were selected a priori based on literature, biological plausibility, and their potential role as confounders, rather than on statistical significance alone. Covariates included age, sex, education, body mass index, waist circumference, tobacco use, and alcohol consumption. Missing data for laboratory measurements were less than 10%. For these variables, self-reported history was used when available. Missing values were considered missing at random. Pairwise available observations were used in network and regression analyses, so participants were not excluded when information on other conditions was missing. Sensitivity analyses were conducted by applying co-occurrence thresholds of 1% and 2% to assess the robustness of the network structure. Conditions with low prevalence (<5%) were excluded to minimise sparse co-occurrence patterns and unstable clustering. All statistical analyses were performed using R software (version 4.4.3). The igraph package was used for network construction and community detection, while ggplot2 was used for data visualization. A p-value of less than 0.05 was considered statistically significant.

### Ethics statement

The study was approved by the Institutional Ethics Committee of Sree Chitra Tirunal Institute for Medical Sciences and Technology, Trivandrum (Approval No. SCT/IEC/1511/DEC-2019). However, data collection was delayed due to the COVID-19 pandemic. We collected data between September 2022 and July 2024. Necessary permissions were obtained from the Department of Scheduled Tribes Development, Kerala Forests and Wildlife Department, and other relevant local administrative authorities. All eligible individuals were approached in their households, and written informed consent was obtained from each participant in their local language. The consent form described the nature of the study, the voluntary nature of participation, confidentiality of information, and the right to withdraw at any time without penalty. For participants who were unable to read or write, trained tribal promoters assisted in explaining the consent process in culturally appropriate terms. In such cases, consent was documented using thumb impressions in the presence of an independent witness, ensuring that ethical standards for informed participation were maintained throughout the study.

The study findings were shared with participants, tribal communities, and policymakers through community feedback sessions and policy briefs, promoting evidence-based decision-making.

### Role of the funding source

The funders had no role in study design, data collection, data analysis, interpretation, writing of the report.

## Results

A total of 2333 individuals participated in the study with mean age 50.0 ± 13.2 years. Women comprised 58.1% and men 41.9% of study population (Table 1). The analysis was stratified by age at 50 years cut-off, majority (48%) of adults older than 50 years reported no formal schooling. Tobacco use was more common in the older age group (59% vs. 45%), whereas alcohol consumption was similar (≈24%). Overweight and obesity (BMI > 23 kg/m^2^) predominated in both groups, but underweight (18% vs. 13%) and high waist circumference (26% vs. 23%) were slightly more prevalent after the age of 50 years.

**Table 1:** Sociodemographic, behavioural, clinical, and comorbidity characteristics of the study population (N = 2333).

Variables	≤50 years (N = 1323)	>50 years (N = 1010)	Total (N = 2333)	p value
Sex, n (%)				0.80
Female	766 (57.9%)	590 (58.4%)	1356 (58.1%)	
Male	557 (42.1%)	420 (41.6%)	977 (41.9%)	
Educational status, n (%)^a^				<0.0001
No formal schooling	212 (16.0%)	489 (48.4%)	701 (30.1%)	
Till Secondary school	666 (50.4%)	333 (33.0%)	999 (42.9%)	
Secondary school & above	443 (33.5%)	188 (18.6%)	631 (27.1%)	
Behavioural risk factors				
Tobacco usage, n (%)	596 (45.0%)	592 (58.6%)	1188 (50.9%)	<0.0001
Alcohol consumption, n (%)	323 (24.4%)	237 (23.5%)	560 (24.0%)	0.60
BMI, n (%)^a^				0.026
Normal (18.5–22.9)	497 (41.8%)	372 (40.7%)	869 (41.3%)	
Underweight (<18.5)	160 (13.4%)	168 (18.4%)	328 (15.6%)	
Overweight & obese (≥23)	533 (44.8%)	375 (41.0%)	908 (43.1%)	
Waist circumference, n (%)^a^				0.077
High (Male > 90 cm, Female > 80 cm)	271 (23.0%)	241 (26.3%)	512 (24.4%)	
Normal	909 (77.0%)	675 (73.7%)	1584 (75.6%)	

a Missing data reported.

Participants older than 50 years had higher prevalences of hypertension (70.8% vs. 35.4%), diabetes (42.5% vs. 24.0%), and chronic kidney disease (33.0% vs. 14.5%) compared with younger than or equal to 50 years (Table 2). The prevalence of anaemia remained similar across age groups (42 vs. 44%).

**Table 2:** Prevalence of comorbidities.

Variables	≤50 years (N = 1323)	>50 years (N = 1010)	Total (N = 2333)	p value
Hypertension, n (%)	469 (35.4%)	715 (70.8%)	1184 (50.8%)	<0.0001
Diabetes, n (%)^a^	288 (24.0%)	408 (42.5%)	696 (32.2%)	<0.0001
Anaemia, n (%)^a^	497 (41.8%)	407 (44.4%)	904 (42.9%)	0.23
Chronic kidney disease, n (%)^a^	166 (14.5%)	293 (33.0%)	459 (22.6%)	<0.0001
Heart disease, n (%)^a^	40 (3.0%)	123 (12.4%)	163 (7.0%)	<0.0001
Chronic respiratory disease, n (%)	71 (5.4%)	177 (17.5%)	248 (10.6%)	<0.0001
Cancer, n (%)^a^	15 (1.1%)	12 (1.2%)	27 (1.2%)	0.87
Sickle cell anaemia, n (%)^a^	15 (1.2%)	4 (0.4%)	19 (0.9%)	0.053
Arthritis, n (%)^a^	77 (5.9%)	185 (18.7%)	262 (11.4%)	<0.0001
Epilepsy, n (%)^a^	23 (1.8%)	18 (1.9%)	41 (1.8%)	0.90
Mental disorder, n (%)^a^	20 (1.6%)	18 (1.9%)	38 (1.7%)	0.56
Depression, n (%)	84 (6.3%)	137 (13.6%)	221 (9.5%)	<0.0001
Anxiety, n (%)	50 (3.8%)	56 (5.5%)	106 (4.5%)	0.042

a Missing data reported, n (%): diabetes mellitus 174 (7.5%), anaemia 228 (9.8%), chronic kidney disease 304 (13.0%), heart disease 19 (0.8%), cancer 46 (2.0%), sickle-cell anaemia 175 (7.5%), arthritis.

**Fig. 2:**
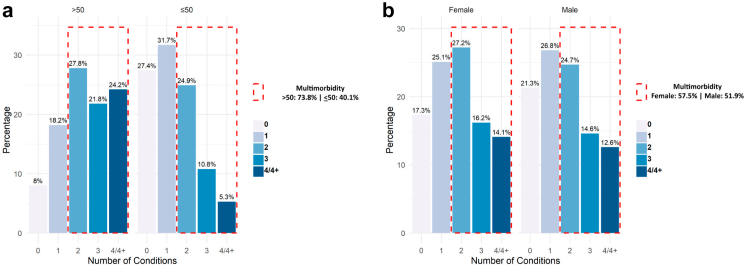
Prevalence of multimorbidity by age group (a) and sex (b). Each bar represents the proportion of comorbidities which is highlighted by red dotted line according to age group and sex.

The overall prevalence of multimorbidity was 55.2% (95% CI: 52.7–57.6). Among participants older than 50 years, 73.8% (95% CI: 70.3–76.9), and among those younger than or equal to 50 years, it was 40.1% (95% CI: 37.8–44.2) (Fig. 2A). Among females, the rate was 57.5% (95% CI: 54.3–60.7), and among males, it was 51.9% (95% CI: 48.1–55.7) (Fig. 2B). The age-standardised prevalence of multimorbidity was 51.2% (95% CI: 49.2%–53.2%), with a relatively higher prevalence among women (53.5%, 95% CI: 51.5%–55.5%) compared to men (48.1%, 95% CI: 46.1%–50.1%).

**Fig. 3:**
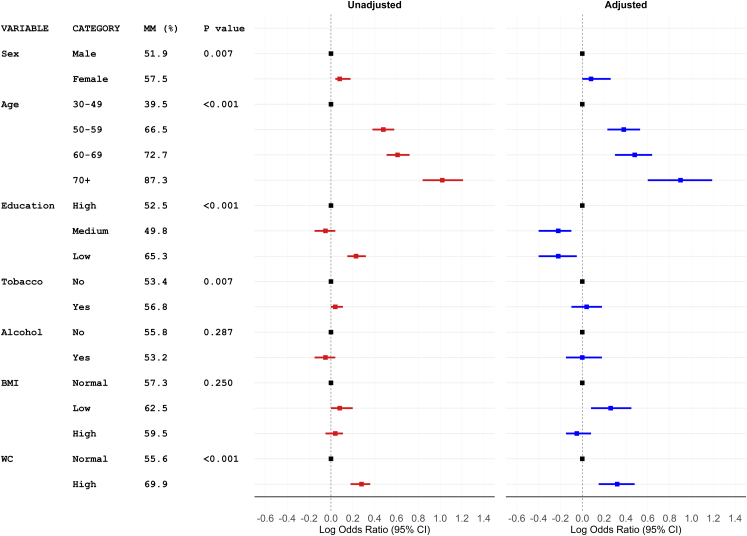
Factors associated with multimorbidity by crude and adjusted models. Reference categories were male for sex, 30–49 years for age, high education, no tobacco use, no alcohol use, and normal body mass index (18.5–22.9) and waist circumference (>90 cm for men and >80 cm for women).

A clear age-related gradient was observed, with prevalence rising from 39.5% (95% CI: 36.2–42.9) among participants 30–49 years of age to 87.3% (95% CI: 80.7–91.9) among those 70 years and above (Fig. 3). After controlling for potential confounders, older age remained a strong predictor of multimorbidity (Fig. 3). Compared with participants between 30 and 49 years of age, the adjusted odds ratios (AOR) were 2.4 (95% CI: 1.7–3.4) for 50–59 years, 3.0 (95% CI: 2.0–4.4) for 60–69 years, and 8.0 (95% CI: 4.0–15.6) for 70 years and above. While the crude prevalence was slightly higher among females (57.5%) compared with males (51.9%), this difference was not statistically significant after adjustment for age, education, behavioural, and clinical variables (AOR = 1.2, 95% CI: 1.0–1.8, p = 0.172) (Fig. 3).

In the multivariable analysis, participants with medium or low education levels had a lower likelihood of multimorbidity compared with those having higher education (AOR = 0.6, 95% CI: 0.4–0.8 and AOR = 0.6, 95% CI: 0.4–0.9, respectively). Tobacco and alcohol use did not show significant associations with multimorbidity. Among anthropometric measures, both low BMI (AOR = 1.8 compared to normal BMI, 95% CI: 1.2–2.8, p = 0.003) and abdominal obesity (AOR = 2.1 compared to normal waist circumference, 95% CI: 1.4–3.0, p < 0.001) were independently associated with multimorbidity (Fig. 3).

**Fig. 4:**
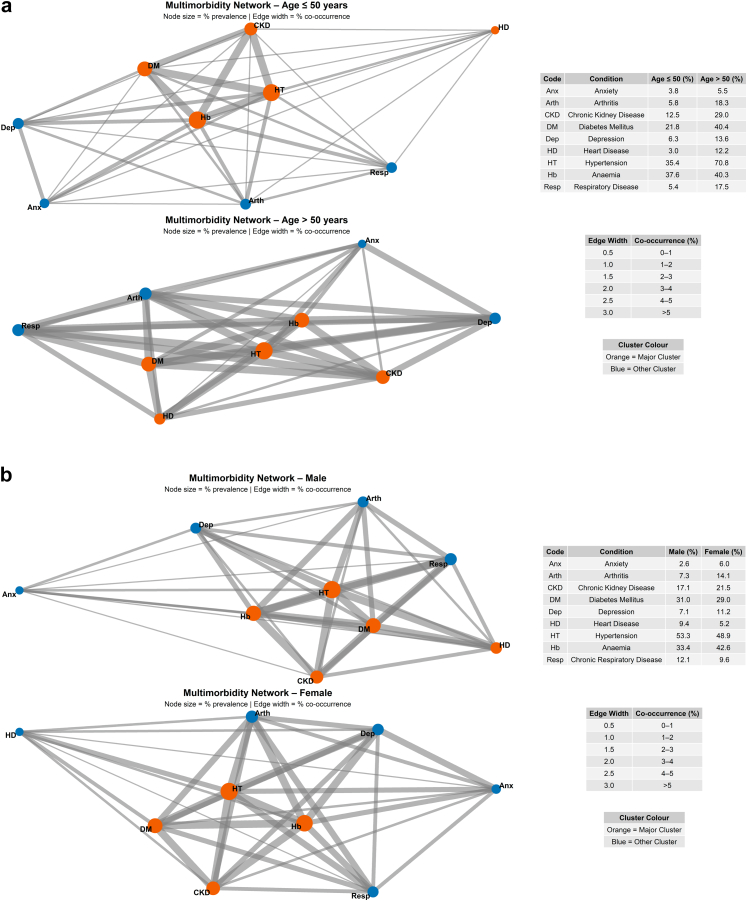
Multimorbidity network using the Louvain method for age group (a) and sex (b). Nodes represent chronic conditions and edges represent co-occurrence between conditions, weighted by joint prevalence. Colours indicate clusters identified using the Louvain community detection algorithm, with orange representing the major cluster and blue representing the minor cluster.

In the multimorbidity network for adults (≤50 and >50 years of age) (Fig. 4A), the largest cluster, identified using the Louvain community detection method, comprised cardio-metabolic conditions such as hypertension, diabetes mellitus, chronic kidney disease, anaemia, and heart disease. A similar cardio-metabolic cluster was also observed when the analysis was stratified by sex (Fig. 4B). Similar clustering patterns were observed in sensitivity analyses using Jaccard-based networks (Online supplement, Supplementary Fig. S1A and B).

The multimorbidity networks included nine chronic conditions represented as nodes and 36 edges representing disease co-occurrence pairs (Online supplement, Supplementary Tables S5–S12). This corresponds to the maximum number of possible edges for a network of nine nodes, resulting in fully connected networks with a density and clustering coefficient of 1.0 and a network diameter of 1 (Online supplement, Supplementary Tables S6 and S10). Two clusters of co-occurring conditions were identified, comprising a cardiometabolic cluster (hypertension, diabetes mellitus, anaemia, heart disease, and chronic kidney disease) and a second cluster including chronic respiratory disease, arthritis, depression, and anxiety (Online supplement, Supplementary Tables S7 and S11). This cluster structure was consistent across age (Online supplement, Supplementary Tables S6 and S7) and sex strata (Online supplement, Supplementary Tables S8–S12), with similar disease composition (Online supplement, Supplementary Tables S6 and S10) and cluster sizes (Online supplement, Supplementary Tables S9 and S13), observed in stratified analyses. Increasing the co-occurrence threshold progressively reduced the number of edges and network density; however, the overall network structure and the ranking of central diseases remained largely unchanged, indicating robustness of the observed multimorbidity patterns. (Online supplement, Supplementary Table S14). Inclusion of low-prevalence conditions did not materially alter the network structure (Online supplement, Supplementary Fig. S2), indicating robustness of the findings.

## Discussion

Our study documents burden of multimorbidity among adults older than or equal to 30 in 18 indigenous tribal populations in Kerala, with more than half of the participants affected. We observed a rise in prevalence of multimorbidity with advancing age, a strong clustering of cardio-metabolic conditions and both low BMI and abdominal obesity were independently associated with multimorbidity. Through comprehensive risk factor profiling, sex-stratified network analyses, and age-standardised prevalence estimates, our study provides insights into the factors associated with multimorbidity among population belonging to tribal communities in Kerala. The high prevalence of multimorbidity observed mirrors the pattern seen in high-income countries. In Kerala, the demographic and epidemiological transition, characterised by an ageing population, has contributed to a shift in increasing disease burden from communicable to non-communicable diseases (NCDs).^28^ There is an increase in proportion of chronic conditions, with NCDs accounting for most deaths and years lived with disability.^28^ Within this context, the high prevalence of multimorbidity found among indigenous communities closely align with global evidence, wherein indigenous groups are approximately twice as likely to develop multiple chronic conditions compared to non-indigenous peers.^10^

Using Louvain method, this study identified a common cluster comprising cardio-metabolic conditions, including hypertension, diabetes, chronic kidney disease, and anaemia. It reflects the interconnectedness of these chronic diseases and highlights potential shared metabolic and behavioural risk factors.^29^^,^^30^ Although we could not assess longitudinal clinical outcomes, these findings are consistent with both Indian and international studies that have highlighted the tendency of cardio-metabolic disorders to co-occur, especially among socially and economically marginalised populations.^31^^,^^32^ Although traditionally marginalised populations may have faced a higher burden of infectious diseases, Kerala’s epidemiological transition has contributed to the dual risk of persistence of infectious diseases and a sharp rise in cardio-metabolic diseases.^4^^,^^33^

High prevalence of multimorbidity among older adults and women is reported in India, a pattern that is mirrored in our findings.^34^ Notably, the similar values of crude and age-standardised prevalence observed in our study highlight that increased multimorbidity cannot be linked solely to the age distribution of the sample population. This conclusion is reinforced by results from the National Family Health Survey (NFHS-5), which demonstrate that state-wise age-standardised multimorbidity varies significantly,^35^ highlighting the role of factors beyond age, such as socioeconomic status, access to care, and education.

A lower BMI was associated with multimorbidity in this population, a pattern that may initially appear paradoxical, considering the well-known relationship between higher adiposity and metabolic disease. This association likely reflects underlying nutritional deficiencies and broader social and biological vulnerabilities that are common in the indigenous communities. Persistent undernutrition and sustained energy deficiency leads to loss of skeletal muscle, which in turn impairs insulin-mediated glucose uptake and promotes abnormalities in glucose homeostasis.^36^, ^37^, ^38^ Additionally, exposure to undernutrition and infection during early life can induce long-term metabolic programming, predisposing individuals to cardiometabolic disorders even at relatively low body weight.^39^ In contrast, higher waist circumference, reflecting central or abdominal adiposity, was independently associated with multimorbidity. Central fat accumulation is considered one of the core features of metabolic dysregulation.^40^ It is linked to insulin resistance, atherogenic dyslipidaemia, elevated blood pressure, and chronic low-grade systemic inflammation, each of which promotes the clustering of multiple cardio-metabolic conditions.^41^^,^^42^ Collectively, these results indicate that both ends of the body composition spectrum, low BMI related to undernutrition and excess central adiposity, may coexist within populations undergoing rapid socio-economic and nutritional transition, and that each could operate through distinct pathophysiological pathways to increase the burden of chronic multimorbidity.

While our study was conducted among tribal population in Kerala, the patterns of multimorbidity observed, particularly the dominance of cardiometabolic clusters and the coexistence of undernutrition with central adiposity, are consistent with findings from other parts of India and from studies conducted globally in low- and middle-income countries. Previous multi-country analyses have reported similar high prevalence of multimorbidity among socioeconomically disadvantaged population, often driven by chronic cardiometabolic conditions.^2^^,^^43^ Preventing and managing multimorbidity among tribal and socioeconomically disadvantaged groups require integrated multi-level strategies with re-organisation of primary care.^44^ Our findings on high prevalence of multimorbidity in the indigenous tribal population of Kerala underscore the need for early detection and integrated care among tribal populations, aligned with NP-NCD and Ayushman Bharat initiatives, and highlight the importance of addressing NCDs.^12^

Though the study systematically examined the prevalence and associated factors of multimorbidity among tribal population in Kerala, including participants from 18 distinct tribal groups, there are several limitations. The study’s cross-sectional design limits the ability to infer causal relationships, and observed associations should not be interpreted as risk factors. Although the sampling strategy aimed to capture diversity across the population by including 18 prominent tribal groups, the findings may not fully represent younger adults (<30 years) or all tribal communities in the state. Kerala has many other tribes which were not included in this study due to logistical constraints, particularly among smaller and more dispersed groups. The age threshold aligns with the Government of India’s National Programme for Prevention & Control of Non-Communicable Diseases (NP-NCD), which recommends screening from 30 years onward due to the substantially increased risk of lifestyle-related chronic conditions. Our study focused on non-communicable chronic conditions, and infectious diseases were excluded due to logistical and regulatory constraints in the tribal setting, including the need for additional governmental approvals and laboratory-based confirmation. Moreover, the episodic nature of many infectious conditions and potential underdiagnosis may limit their reliable assessment in cross-sectional surveys. Diagnosis of chronic conditions was based on single-time measures and self-reports, which may have introduced misclassification. Field measurements, such as cardio-metabolic markers, were performed using validated point-of-care devices rather than laboratory assays, potentially leading to measurement errors. Occasional logistical challenges and participant noncompliance with fasting led to incomplete data for some individuals. Furthermore, data on environmental, occupational, and genetic risk factors were not captured, limiting a complete assessment of all determinants.

Our study shows that one in two adults (>30 years) among tribal population in Kerala are affected by multimorbidity, with higher proportion among older individuals (≥50 years), women, individuals with low educational attainment, low BMI, and abdominal obesity. A cardio-metabolic cluster was identified involving hypertension, diabetes, chronic kidney disease, and anaemia highlighting strong interconnection among these conditions. Our findings have important implications for public health programs such as NP-NCD and Ayushman Bharat, which currently focus on individual diseases. There is a need to strengthen the NCD framework to address multimorbidity through integrated, patient-centred approaches that prioritise vulnerable populations. Community health workers and local health promoters serve a vital role in identifying individuals at risk and ensuring they access appropriate care. This includes coordinated screening for common clusters of conditions, continuity of care across primary and secondary levels, and improved access to essential diagnostics and medications in underserved areas. Social support initiatives, including the provision of subsidised medications, diagnostic services, and transportation to healthcare facilities, should be targeted towards individuals with limited financial resources and educational opportunities. Multisectoral collaboration addressing social determinants such as nutrition, education, and access to care will be critical to reduce the burden of multimorbidity and improve health equity in tribal populations.

## Contributors

PJ, MV, JVT, and SG conceived the study. PJ and SI coordinated and supervised data collection, had full access to the data, were responsible for data verification, and contributed to data analysis, visualisation, initial interpretation, and drafting of the manuscript. All authors critically reviewed the manuscript and approved the final version for publication.

## Data sharing statement

The data supporting this study’s findings are available upon reasonable request and should be addressed to the corresponding author.

## Declaration of interests

We declare no competing interests.

## References

[1] Chowdhury S.R., Das D.C., Sunna T.C., Beyene J., Hossain A. (2023). Global and regional prevalence of multimorbidity in the adult population in community settings: a systematic review and meta-analysis. eClinicalMedicine.

[2] Afshar S., Roderick P.J., Kowal P., Dimitrov B.D., Hill A.G. (2015). Multimorbidity and the inequalities of global ageing: a cross-sectional study of 28 countries using the World Health Surveys. BMC Public Health.

[3] Zanwar P.P., Taylor R., Hill-Jarrett T.G. (2024). Characterizing multimorbidity prevalence and adverse outcomes in ethnically and culturally diverse sub-populations in India: gaps, opportunities, and future directions. Int J Environ Res Publ Health.

[4] Narain J.P. (2019). Health of tribal populations in India: how long can we afford to neglect?. Indian J Med Res.

[5] Negi D.P., Abdul Azeez E.P. (2021). Diminishing traditional methods and inaccessible modern healthcare: the dilemma of tribal health in India. J Health Res.

[6] Puri P., Pati S. (2022). Exploring the linkages between non-communicable disease multimorbidity, health care utilization and expenditure among Aboriginal older adult population in India. Int J Public Health.

[7] Kaur P., Borah P.K., Uike P.V. (2025). Non-communicable diseases as a major contributor to deaths in 12 tribal districts in India. Indian J Med Res.

[8] Linda A.I., Pal D., Murmu N., Taywade M. (2024). Health of tribal population in India: a glimpse of the current scenario. Curr Med Issues.

[9] Sahu M., Kujur A., Venugopal V. (2024). Tribal health: a public health exigency and road map to future. Indian J Community Med.

[10] Shahunja K., Ushula T.W., Hussain M.A., Pati S., Mamun A.A. (2024). Multimorbidity among the Indigenous population: a systematic review and meta-analysis. Ann Epidemiol.

[11] AK K., Kshatri J.S., van den Akker M., Hussain M.A., Bhattacharya H., Pati S. (2025). Epidemiology of multimorbidity among indigenous adults: insights from a large-scale population survey of 53 different indigenous groups in East India. Glob Epidemiol.

[12] Ministry of Health & Family Welfare, Government of India (2023). https://www.mohfw.gov.in/sites/default/files/NP-NCD%20Operational%20Guidelines_0.pdf.

[13] Ahamad V., Mohammad R., Pal A.K., Chouhan K.R. (2025). Multimorbidity and its association with health-related quality of life among older adults in India: a cross-sectional analysis of LASI wave-1. BMC Geriatr.

[14] Heatherton T.F., Kozlowski L.T., Frecker R.C., Fagerstrom K.O. (1991). The fagerström test for nicotine dependence: a revision of the fagerstrom tolerance questionnaire. Br J Addict.

[15] Saunders J.B., Aasland O.G., Babor T.F., De La Fuente J.R., Grant M. (1993). Development of the alcohol use disorders identification test (AUDIT): WHO collaborative project on early detection of persons with harmful alcohol Consumption-II. Addiction.

[16] Bull F.C., Maslin T.S., Armstrong T. (2009). Global physical activity questionnaire (GPAQ): nine country reliability and validity study. J Phys Activ Health.

[17] Kroenke K., Spitzer R.L., Williams J.B.W. (2001). The PHQ-9. J Gen Intern Med.

[18] Spitzer R.L., Kroenke K., Williams J.B.W., Löwe B. (2006). A brief measure for assessing generalized anxiety disorder: the GAD-7. Arch Intern Med.

[19] Duncan P., Murphy M., Man M.S., Chaplin K., Gaunt D., Salisbury C. (2020). Development and validation of the multimorbidity treatment burden questionnaire (MTBQ). BMJ Open.

[20] Skou S.T., Mair F.S., Fortin M. (2022). Multimorbidity. Nat Rev Dis Primers.

[21] Chobanian A.V., Bakris G.L., Black H.R. (2003). Seventh report of the joint national committee on prevention, detection, evaluation, and treatment of high blood pressure. Hypertension.

[22] American Diabetes Association (2020). Classification and diagnosis of diabetes: standards of medical care in Diabetes—2021. Diabetes Care.

[23] Kidney Disease: Improving Global Outcomes (KDIGO) CKD Work Group (2024). KDIGO 2024 clinical practice guideline for the evaluation and management of chronic kidney disease. Kidney Int.

[24] Balarajan Y., Ramakrishnan U., Özaltin E., Shankar A.H., Subramanian S. (2011). Anaemia in low-income and middle-income countries. Lancet.

[25] WHO Western Pacific Region (WPRO) (2000). http://iris.wpro.who.int/handle/10665.1/5379.

[26] von Elm E., Altman D.G., Egger M. (2007). The strengthening the reporting of observational studies in epidemiology (STROBE) statement: guidelines for reporting observational studies. Ann Intern Med.

[27] Ferris J.K., Fiedeldey L.K., Kim B. (2025). A systematic review and meta-analysis of disease clusters in multimorbidity. Nat Commun.

[28] Prabhakaran D., Jeemon P., Sharma M. (2018). The changing patterns of cardiovascular diseases and their risk factors in the states of India: the Global Burden of Disease Study 1990–2016. Lancet Global Health.

[29] Raimundo M., Lopes J.A. (2011). Metabolic syndrome, chronic kidney disease, and cardiovascular disease: a dynamic and life-threatening triad. Cardiol Res Pract.

[30] Mao D., Mu J., Li Y. (2025). Network-based machine learning reveals cardiometabolic multimorbidity patterns and modifiable lifestyle factors: a community-focused analysis of NHANES 2015–2018. BMC Public Health.

[31] Anand S.S., Kandasamy S., Marchand M. (2025). Reducing inequalities in cardiovascular disease: focus on marginalized populations considering ethnicity and race. Lancet Reg Health Eur.

[32] Jana A., Chattopadhyay A. (2022). Prevalence and potential determinants of chronic disease among elderly in India: Rural-Urban perspectives. PLoS One.

[33] Thomas B.E., Adinarayanan S., Manogaran C., Swaminathan S. (2025). Pulmonary tuberculosis among tribals in India: a systematic review & meta-analysis. Indian J Med Res.

[34] Sinha A., Kerketta S., Ghosal S., Kanungo S., Lee J.T., Pati S. (2022). Multimorbidity and complex multimorbidity in India: findings from the 2017–2018 longitudinal ageing study in India (LASI). Int J Environ Res Publ Health.

[35] Prenissl J., Neve J.W.D., Sudharsanan N. (2022). Patterns of multimorbidity in India: a nationally representative cross-sectional study of individuals aged 15 to 49 years. PLoS Glob Publ Health.

[36] Shetty P.S. (1990). Physiological mechanisms in the adaptive response of metabolic rates to energy restriction. Nutr Res Rev.

[37] Iorember F.M. (2018). Malnutrition in chronic kidney disease. Front Pediatr.

[38] James W.P.T., Coore H.G. (1970). Persistent impairment of insulin secretion and glucose tolerance after Malnutrition 1. Am J Clin Nutr.

[39] Fall C.H.D., Kumaran K. (2019). Metabolic programming in early life in humans. Phil Trans Biol Sci.

[40] Cnop M., Landchild M.J., Vidal J. (2002). The concurrent accumulation of intra-abdominal and subcutaneous fat explains the association between insulin resistance and plasma leptin concentrations: Distinct metabolic effects of two fat compartments. Diabetes.

[41] Hwang Y.C., Fujimoto W.Y., Hayashi T., Kahn S.E., Leonetti D.L., Boyko E.J. (2016). Increased visceral adipose tissue is an independent predictor for future development of atherogenic dyslipidemia. J Clin Endocrinol Metab.

[42] de Souza R.J., Pigeyre M.E., Schulze K.M. (2025). Visceral adipose tissue and hepatic fat as determinants of carotid atherosclerosis. Commun Med.

[43] Garin N., Koyanagi A., Chatterji S. (2016). Global multimorbidity patterns: a cross-sectional, population-based, multi-country study. J Gerontol A Biol Sci Med Sci.

[44] Lekha T.R., Joseph L., Sasidharan N.V. (2025). Healthcare providers’ perspectives on the organization of health services to manage people with multiple long-term conditions in primary care settings in Kerala, India: a qualitative exploratory study. Front Public Health.

