# Supplementary material for: Multimorbidity among indigenous tribal communities in Kerala, India: a cross-sectional study (2022–2024)
Source: Lancet Reg Health Southeast Asia. 2026 May 14;49:100781. doi: 10.1016/j.lansea.2026.100781 (PMC13196445; doi:10.1016/j.lansea.2026.100781)

**Table S1. STROBE Checklist**

|  | **Item Description** | **Location (or reason for not reporting)** |
| --- | --- | --- |
| **Title and abstract** |  |  |
| [1a. Indicate the study’s design](https:/resources.equator-network.org/reporting-guidelines/strobe/items/title-abstract-indicate-study-design.html) | Indicate the study’s design with a commonly used term in the title or the abstract. | Title- Cross sectional study |
| [1b. Abstract](https:/resources.equator-network.org/reporting-guidelines/strobe/items/abstract.html) | Provide in the abstract an informative and balanced summary of what was done and what was found. | Abstract- Methods- 1^st^ line |
| **Introduction** |  |  |
| [2. Background/rationale](https:/resources.equator-network.org/reporting-guidelines/strobe/items/background-rationale.html) | Explain the scientific background and rationale for the investigation being reported. | Abstract -Introduction  Introduction-1-3 paragraph |
| [3. Objectives](https:/resources.equator-network.org/reporting-guidelines/strobe/items/objectives.html) | State specific objectives, including any prespecified hypotheses. | Abstract -Introduction-Last Line  Introduction-5^th^ Paragraph |
| **Methods** |  |  |
| [4. Study design](https:/resources.equator-network.org/reporting-guidelines/strobe/items/study-design.html) | Present key elements of study design early in the paper. | Abstract- Methods  Methods-  Study Design and Setting |
| [5. Setting](https:/resources.equator-network.org/reporting-guidelines/strobe/items/setting.html) | Describe the setting, locations, and relevant dates, including periods of recruitment, exposure, follow-up, and data collection. | Abstract- Methods  Methods-  Study Design and Setting |
| [6a. Eligibility criteria](https:/resources.equator-network.org/reporting-guidelines/strobe/items/eligibility-criteria.html) | **Cohort study:** Give the eligibility criteria, and the sources and methods of selection of participants. Describe methods of follow-up. **Case-control study:** Give the eligibility criteria, and the sources and methods of case ascertainment and control selection. Give the rationale for the choice of cases and controls. **Cross-sectional study:** Give the eligibility criteria, and the sources and methods of selection of participants. | Abstract- Methods  Methods- Study Design and Setting. 2^nd^ and 3^rd^ Line.  Methods- Sample Size Calculation and Sampling Technique- 2^nd^ paragarph |
| [6b. Matching criteria](https:/resources.equator-network.org/reporting-guidelines/strobe/items/matching-criteria.html) | **Cohort study:** For matched studies, give matching criteria and number of exposed and unexposed. **Case-control study:** For matched studies, give matching criteria and the number of controls per case. | Not applicable to this study |
| [7. Variables](https:/resources.equator-network.org/reporting-guidelines/strobe/items/variables.html) | Clearly define all outcomes, exposures, predictors, potential confounders, and effect modifiers. Give diagnostic criteria, if applicable. | Methods- Measurements and Assessments- 1^st^ and 2^nd^ paragraph.  Methods- Operational definitions |
| [8. Data sources / measurement](https:/resources.equator-network.org/reporting-guidelines/strobe/items/data-sources-measurement.html) | For each variable of interest give sources of data and details of methods of assessment (measurement). Describe comparability of assessment methods if there is more than one group. | Methods- Study Design and Setting. 2^nd^ and 3^rd^ Line.  Methods- Operational definitions and CKD Assessment |
| [9. Bias](https:/resources.equator-network.org/reporting-guidelines/strobe/items/bias.html) | Describe any efforts to address potential sources of bias. | Not applicable |
| [10. Study size](https:/resources.equator-network.org/reporting-guidelines/strobe/items/study-size.html) | Explain how the study size was arrived at. | Methods-Sample Size Calculation and Sampling Technique |
| [11. Quantitative variables](https:/resources.equator-network.org/reporting-guidelines/strobe/items/quantitative-variables.html) | Explain how quantitative variables were handled in the analyses. If applicable, describe which groupings were chosen, and why. | Methods- Statistical Analysis |
| [12a. Statistical methods](https:/resources.equator-network.org/reporting-guidelines/strobe/items/statistical-methods-description.html) | Describe all statistical methods, including those used to control for confounding. | Methods- Statistical Analysis |
| [12b. Statistical methods – subgroups and interactions](https:/resources.equator-network.org/reporting-guidelines/strobe/items/statistical-methods-subgroups-interactions.html) | Describe any methods used to examine subgroups and interactions. | Methods- Statistical Analysis |
| [12c. Statistical methods – missing data](https:/resources.equator-network.org/reporting-guidelines/strobe/items/statistical-methods-missing-data.html) | Explain how missing data were addressed. | Methods- Statistical Analysis |
| [12di. Statistical methods – loss to follow-up](https:/resources.equator-network.org/reporting-guidelines/strobe/items/statistical-methods-loss-to-follow-up.html) | **Cohort study:** If applicable, describe how loss to follow-up was addressed. | Not applicable to this study |
| [12dii. Statistical methods – matching cases and controls](https:/resources.equator-network.org/reporting-guidelines/strobe/items/statistical-methods-matching-cases-controls.html) | **Case-control study:** If applicable, explain how matching of cases and controls was addressed. | Not applicable to this study |
| [12diii. Statistical methods – sampling strategy](https:/resources.equator-network.org/reporting-guidelines/strobe/items/statistical-methods-analytical-methods-sampling-strategy.html) | **Cross-sectional study:** If applicable, describe analytical methods taking account of sampling strategy. | Methods- Statistical Analysis |
| [12e. Statistical methods – sensitivity analyses](https:/resources.equator-network.org/reporting-guidelines/strobe/items/statistical-methods-sensitivity-analyses.html) | Describe any sensitivity analyses. | Results, Supplementary File |
| **Results** |  |  |
| [13a. Participant numbers](https:/resources.equator-network.org/reporting-guidelines/strobe/items/participants-numbers.html) | Report the numbers of individuals at each stage of the study—e.g., numbers potentially eligible, examined for eligibility, confirmed eligible, included in the study, completing follow-up, and analysed; Consider use of a flow diagram. | Results -1^st^ paragraph |
| [13b. Participants – non-participation](https:/resources.equator-network.org/reporting-guidelines/strobe/items/participants-non-participation.html) | Give reasons for non-participation at each stage. | Trial flow chart |
| [13c. Participants – flow diagram](https:/resources.equator-network.org/reporting-guidelines/strobe/items/participants-flow-diagram.html) | Consider use of a flow diagram. | Trail flow chart Figure 1 |
| [14a. Descriptive data – participant characteristics](https:/resources.equator-network.org/reporting-guidelines/strobe/items/descriptive-data-participant-characteristics.html) | Give characteristics of study participants (e.g., demographic, clinical, social) and information on exposures and potential confounders. Present the information in a table. | Results -1^st^ paragraph  **Table 1.**  *Sociodemographic, behavioural, clinical, and comorbidity characteristics of the study population (N = 2,333)* |
| [14b. Descriptive data – missing data](https:/resources.equator-network.org/reporting-guidelines/strobe/items/descriptive-data-missing-data.html) | Indicate the number of participants with missing data for each variable of interest. | Table 2- Footer note |
| [14c. Descriptive data – follow-up time](https:/resources.equator-network.org/reporting-guidelines/strobe/items/descriptive-data-follow-up-time.html) | **Cohort study:** Summarise follow-up time—e.g., average and total amount. | Not applicable to this study |
| [15. Outcome data](https:/resources.equator-network.org/reporting-guidelines/strobe/items/outcome-data.html) | **Cohort study:** Report numbers of outcome events or summary measures over time. **Case-control study:** Report numbers in each exposure category, or summary measures of exposure. **Cross-sectional study:** Report numbers of outcome events or summary measures. | Figure 1. Factors associated with multimorbidity |
| [16a. Main results](https:/resources.equator-network.org/reporting-guidelines/strobe/items/main-results.html) | Give unadjusted estimates and, if applicable, confounder-adjusted estimates and their precision (e.g., 95% confidence intervals). Make clear which confounders were adjusted for and why they were included. | Results- Participant Characteristics, Prevalence of Multimorbidity, Multivariable Analysis of Multimorbidity Determinants  **Table 2.**  *Comparison of sociodemographic, behavioural, clinical, and comorbidity characteristics among patients with and without Multimorbidity* |
| [16b. Main results – category boundaries](https:/resources.equator-network.org/reporting-guidelines/strobe/items/main-results-category-boundaries.html) | Report category boundaries when continuous variables were categorised. | Results- Prevalence of Multimorbidity, Multivariable Analysis of Multimorbidity Determinants |
| [16c. Main results – risk](https:/resources.equator-network.org/reporting-guidelines/strobe/items/main-results-risk.html) | If relevant, consider translating estimates of relative risk into absolute risk for a meaningful time period. | Results- Prevalence of Multimorbidity, Multivariable Analysis of Multimorbidity Determinants |
| [17. Other analyses](https:/resources.equator-network.org/reporting-guidelines/strobe/items/other-analyses.html) | Report other analyses done—e.g., analyses of subgroups and interactions, and sensitivity analyses. | Result |
| **Discussion** |  |  |
| [18. Key results](https:/resources.equator-network.org/reporting-guidelines/strobe/items/key-results.html) | Summarise key results with reference to study objectives. | Discussion-1^st^ paragraph |
| [19. Limitations](https:/resources.equator-network.org/reporting-guidelines/strobe/items/limitations.html) | Discuss limitations of the study, taking into account sources of potential bias or imprecision. Discuss both direction and magnitude of any potential bias. | Discussion- Strengths and Limitations-2^nd^ paragraph |
| [20. Interpretation](https:/resources.equator-network.org/reporting-guidelines/strobe/items/interpretation.html) | Give a cautious overall interpretation considering objectives, limitations, multiplicity of analyses, results from similar studies, and other relevant evidence. | Discussion- 1- 4 paragraph |
| [21. Generalisability](https:/resources.equator-network.org/reporting-guidelines/strobe/items/generalisability.html) | Discuss the generalisability (external validity) of the study results. | Discussion- Strengths and Limitations-3^rd^ Line |
| **Other information** |  |  |
| [22. Funding](https:/resources.equator-network.org/reporting-guidelines/strobe/items/funding.html) | Give the source of funding and the role of the funders for the present study and, if applicable, for the original study on which the present article is based. | Funding |

**Table S2. Summary of biochemical and anthropometric measurements employed in the study**

| **Measurement method / device** | **Standards / Certification** |
| --- | --- |
| Seca 213 stadiometer (height) | CE certified |
| Omron HN-289 weighing scale (weight) | CE certified |
| Seca 201 tape (waist circumference) | CE, ISO compliant |
| Omron HEM-7120 BP monitor | FDA-approved; validated by ESH/AAMI/BHS |
| Aina glucose monitor | FDA 510(k), CE certified |
| Quik-Check Hb / Aina Hb systems | WHO-prequalified, FDA-approved, CE marked |
| StatSensor Xpress (capillary creatinine) | FDA-approved, CLIA-waived, CE marked |
| Indirect ISE method (urinary sodium, potassium) | NABL-accredited laboratory |
| Jaffe kinetic assay, IDMS-traceable (urinary creatinine) | NABL-accredited laboratory |
| Immunoturbidimetric assay (urinary albumin) | NABL-accredited laboratory |
| Calculated ACR from certified component assays | Based on validated laboratory methods |

**Table S3: Conditions Included in the Multimorbidity Assessment**

| **Sl. No** | **Condition Code** | **Full Condition Name** | **Method of Assessment** |
| --- | --- | --- | --- |
| 1 | HT | Hypertension | Measured BP ≥140/90 mmHg or medication |
| 2 | DM | Diabetes Mellitus | Fasting/random glucose thresholds or medication |
| 3 | Hb | Anaemia | Hemoglobin measured via point-of-care device |
| 4 | CKD | Chronic Kidney Disease | Measured or documented medical history |
| 5 | Dep | Depression | Self-report using validated screening questions |
| 6 | Anx | Anxiety | Self-report using validated screening questions |
| 7 | HD | Heart Disease | Self-report of physician diagnosis |
| 8 | Resp | Chronic Respiratory Disease | Self-report of physician diagnosis |
| 9 | Arth | Arthritis | Self-report of physician diagnosis |
| 10 | Ca | Cancer | Self-report of physician diagnosis |
| 11 | MentD | Mental Disorders | Self-report or prior clinical diagnosis |
| 12 | SCA | Sickle Cell Anaemia | Documented diagnosis or prior record |
| 13 | Epi | Epilepsy | Documented diagnosis or prior record |

**Table S4: Network metrics and their description**

| **Network Metrics** | **Description** |
| --- | --- |
| **Nodes** | Each chronic condition included in the network (e.g., hypertension, diabetes, CKD). |
| **Edges** | Connections between conditions indicating how often they co-occur in participants. |
| **Diameter** | The longest shortest path between any two conditions in the network; reflects how “spread out” the network is. |
| **Density** | Proportion of possible connections that actually exist; higher density means more conditions tend to co-occur |
| **Clustering Coefficient** | Measures the tendency of conditions to form tightly connected groups (e.g., cardio-metabolic cluster of hypertension, diabetes, CKD). |
| **Centralization** | Indicates whether certain conditions are highly connected compared with others (e.g., a “hub” condition like hypertension). |
| **Modularity** | Quantifies how well the network divides into clusters; higher modularity indicates distinct groups of co-occurring diseases. |
| **Jaccard Index** | measure of similarity between two conditions, defined as the proportion of individuals who have *both* conditions relative to those who have *either* condition.  Values range from **0 to 1**, where:   - **0** indicates no co-occurrence - **1** indicates perfect overlap   J(A,B)= Number with both A and B/ Number with A and B |

**Table S5.** Unadjusted and adjusted associations of sociodemographic and behavioural factors with multimorbidity

| **Variable** | **Category** | **OR (Unadjusted)** | **95% CI (Unadjusted)** | **OR (Adjusted)** | **95% CI (Adjusted)** | **Global p-value** | **N (%)** |
| --- | --- | --- | --- | --- | --- | --- | --- |
| Sex | Male | 1 (Ref) | – | 1 (Ref) | – | 0.007 | 507 (51.9) |
|  | Female | 1.2 | 1.1 – 1.5 | 1.2 | 1.0 – 1.8 |  | 780 (57.5) |
| Age | 30–49 | 1 (Ref) | – | 1 (Ref) | – | <0.001 | 489 (39.5) |
|  | 50–59 | 3.0 | 2.4 – 3.8 | 2.4 | 1.7 – 3.4 |  | 292 (66.5) |
|  | 60–69 | 4.1 | 3.2 – 5.2 | 3.0 | 2.0 – 4.4 |  | 328 (72.7) |
|  | 70+ | 10.5 | 6.9 – 16.1 | 8.0 | 4.0 – 15.6 |  | 178 (87.3) |
| Education | High | 1 (Ref) | – | 1 (Ref) | – | <0.001 | 331 (52.5) |
|  | Medium | 0.9 | 0.7 – 1.1 | 0.6 | 0.4 – 0.8 |  | 498 (49.8) |
|  | Low | 1.7 | 1.4 – 2.1 | 0.6 | 0.4 – 0.9 |  | 458 (65.3) |
| Tobacco | No | 1 (Ref) | – | 1 (Ref) | – | 0.102 | 612 (53.4) |
|  | Yes | 1.1 | 1.0 – 1.3 | 1.1 | 0.8 – 1.5 |  | 675 (56.8) |
| Alcohol | No | 1 (Ref) | – | 1 (Ref) | – | 0.287 | 989 (55.8) |
|  | Yes | 0.9 | 0.7 – 1.1 | 1.0 | 0.7 – 1.5 |  | 298 (53.2) |
| BMI | Normal | 1 (Ref) | – | 1 (Ref) | – | 0.250 | 498 (57.3) |
|  | Low | 1.2 | 1.0 – 1.6 | 1.8 | 1.2 – 2.8 |  | 205 (62.5) |
|  | High | 1.1 | 0.9 – 1.3 | 0.9 | 0.7 – 1.2 |  | 540 (59.5) |
| WC | Normal | 1 (Ref) | – | 1 (Ref) | – | <0.001 | 881 (55.6) |
|  | High | 1.9 | 1.5 – 2.3 | 2.1 | 1.4 – 3.0 |  | 358 (69.9) |

**Table S6. Network metrics for age-stratified multimorbidity networks**

| Age Group | Nodes | Edges | Diameter | Density | Clustering Coefficient | Centralization | Modularity |
| --- | --- | --- | --- | --- | --- | --- | --- |
| ≤50 years | 9 | 36 | 1 | 1.00 | 1.00 | 0 | 0.067 |
| >50 years | 9 | 36 | 1 | 1.00 | 1.00 | 0 | 0.014 |

**Table S7. Diseases in the largest clusters of age-stratified networks**

| Age Group | Cluster | Diseases |
| --- | --- | --- |
| ≤50 years | 1 | Hypertension, Diabetes Mellitus, Anaemia, Heart Disease, Chronic Kidney Disease |
| >50 years | 1 | Hypertension, Diabetes Mellitus, Anaemia, Heart Disease, Chronic Kidney Disease |

**Table S8. Disease cluster membership in age-stratified networks**

| **Disease** | **Code** | **Cluster** | **Age Group** |
| --- | --- | --- | --- |
| Hypertension | HT | 1 | ≤50 |
| Diabetes Mellitus | DM | 1 | ≤50 |
| Anaemia | Hb | 1 | ≤50 |
| Heart Disease | HD | 1 | ≤50 |
| Chronic Kidney Disease | CKD | 1 | ≤50 |
| Chronic Respiratory Disease | Resp | 2 | ≤50 |
| Arthritis | Arth | 2 | ≤50 |
| Depression | Dep | 2 | ≤50 |
| Anxiety | Anx | 2 | ≤50 |
| Hypertension | HT | 1 | >50 |
| Diabetes Mellitus | DM | 1 | >50 |
| Anaemia | Hb | 1 | >50 |
| Heart Disease | HD | 1 | >50 |
| Chronic Kidney Disease | CKD | 1 | >50 |
| Chronic Respiratory Disease | Resp | 2 | >50 |
| Arthritis | Arth | 2 | >50 |
| Depression | Dep | 2 | >50 |
| Anxiety | Anx | 2 | >50 |

**Table S9. Cluster sizes in sex-stratified networks**

| **Age Group** | **Cluster** | **Number of Diseases** |
| --- | --- | --- |
| ≤50 years | 1 | 5 |
| ≤50 years | 2 | 4 |
| >50 years | 1 | 5 |
| >50 years | 2 | 4 |

**Table S10. Network metrics for age-stratified multimorbidity networks**

| **Sex** | **Nodes** | **Edges** | **Diameter** | **Density** | **Clustering Coefficient** | **Centralization** | **Modularity** |
| --- | --- | --- | --- | --- | --- | --- | --- |
| Male | 9 | 36 | 1 | 1.00 | 1.00 | 0 | 0.011 |
| Female | 9 | 36 | 1 | 1.00 | 1.00 | 0 | 0.036 |

**Table S11. Diseases in the largest clusters of sex-stratified networks**

| **Sex** | **Cluster** | **Diseases** |
| --- | --- | --- |
| Male | 1 | Hypertension, Diabetes Mellitus, Anaemia, Heart Disease, Chronic Kidney Disease |
| Female | 2 | Heart Disease, Chronic Respiratory Disease, Arthritis, Depression, Anxiety |

**Table S12. Disease cluster membership in sex-stratified networks**

| **Disease** | **Code** | **Cluster** | **Sex** |
| --- | --- | --- | --- |
| Hypertension | HT | 1 | Male |
| Diabetes Mellitus | DM | 1 | Male |
| Anaemia | Hb | 1 | Male |
| Heart Disease | HD | 1 | Male |
| Chronic Kidney Disease | CKD | 1 | Male |
| Chronic Respiratory Disease | Resp | 2 | Male |
| Arthritis | Arth | 2 | Male |
| Depression | Dep | 2 | Male |
| Anxiety | Anx | 2 | Male |
| Hypertension | HT | 1 | Female |
| Diabetes Mellitus | DM | 1 | Female |
| Anaemia | Hb | 1 | Female |
| Heart Disease | HD | 2 | Female |
| Chronic Kidney Disease | CKD | 1 | Female |
| Chronic Respiratory Disease | Resp | 2 | Female |
| Arthritis | Arth | 2 | Female |
| Depression | Dep | 2 | Female |
| Anxiety | Anx | 2 | Female |

**Table S13. Cluster sizes in sex-stratified networks**

| **Sex** | **Cluster** | **Number of Diseases** |
| --- | --- | --- |
| Male | 1 | 5 |
| Male | 2 | 4 |
| Female | 1 | 4 |
| Female | 2 | 5 |

**Table S14.** Summary of Network Metrics at Different Thresholds

Male Network

| **Threshold** | **Nodes** | **Edges** | **Diameter** | **Density** | **Clustering Coefficient** | **Centralization** | **Modularity** |
| --- | --- | --- | --- | --- | --- | --- | --- |
| No threshold | 9 | 36 | 1 | 1.000 | 1.000 | 0.000 | 0.011 |
| 1% threshold | 9 | 32 | 2 | 0.889 | 0.921 | 0.111 | 0.010 |
| 2% threshold | 9 | 23 | 2 | 0.639 | 0.828 | 0.236 | 0.000 |

Female Network

| **Threshold** | **Nodes** | **Edges** | **Diameter** | **Density** | **Clustering Coefficient** | **Centralization** | **Modularity** |
| --- | --- | --- | --- | --- | --- | --- | --- |
| No threshold | 9 | 36 | 1 | 1.000 | 1.000 | 0.000 | 0.036 |
| 1% threshold | 9 | 34 | 2 | 0.944 | 0.947 | 0.056 | 0.041 |
| 2% threshold | 9 | 28 | 2 | 0.778 | 0.868 | 0.222 | 0.053 |

Increasing the co-occurrence threshold progressively reduced the number of edges and network density; however, the overall network structure and the ranking of central diseases remained largely unchanged, indicating robustness of the observed multimorbidity patterns.

**Table S15. R Analysis Workflow and Sample Details**

| **Step** | **Description** | **Code/Notes** | |
| --- | --- | --- | --- |
| **Data Import** | Load Excel data, ensure numeric | df <- read_excel("path/to/sexMM.xlsx")df <- df %>% mutate(across(-Sex, ~ replace_na(as.numeric(.),0)) |  |
| **Prevalence Calculation** | Compute % prevalence by sex/condition | pivot_longer() + group_by(Sex, Code) + summarise(Percent = round(mean(value)*100,1)) |  |
| **Age-stratified Bar Plots** | Plot % distribution of number of conditions (≤50 vs >50) | ggplot(data_long, aes(...)) + geom_bar() + facet_wrap(~AgeGroup)annotate("rect", ...) for multimorbidity |  |
| **Regression Forest Plots** | ORs (adjusted/unadjusted), p-values, multimorbidity % | ggplot(plot_data, aes(x=OR, y=y, color=Type)) + geom_point() + geom_segment(aes(x=LowerCI, xend=UpperCI)) |  |
| **Jaccard Network (Sex-stratified)** | Create adjacency matrices, compute Jaccard similarity | co_mat <- as.matrix(data[, include_vars])jaccard_mat[i,j] <- intersection/union |  |
| **Network Construction** | Build undirected weighted network, compute Louvain clusters | g <- graph_from_adjacency_matrix(jaccard_mat, mode="undirected", weighted=TRUE)clusters <- cluster_louvain(g) |  |
| **Node Attributes** | Prevalence-based node sizing, cluster color coding | as_tbl_graph(g) %>% mutate(prevalence=..., color=...) |  |
| **Edge Attributes** | Weight proportional to Jaccard % | mutate(edge_width = case_when(...)) |  |
| **Plotting Network** | Node size = prevalence, Edge width = co-occurrence | ggraph(...)+geom_edge_link()+geom_node_point()+geom_node_text() |  |
| **Legends** | Node prevalence, edge width, cluster color | tableGrob() + arrangeGrob() for combined legend |  |
| **Export** | High-res TIFF for manuscript | tiff("file.tiff", width=18, height=10, units="in", res=600)grid.arrange(...)dev.off() |  |

**Figure S1A**. Jaccard-Weighted Multimorbidity Network Analysis by Sex


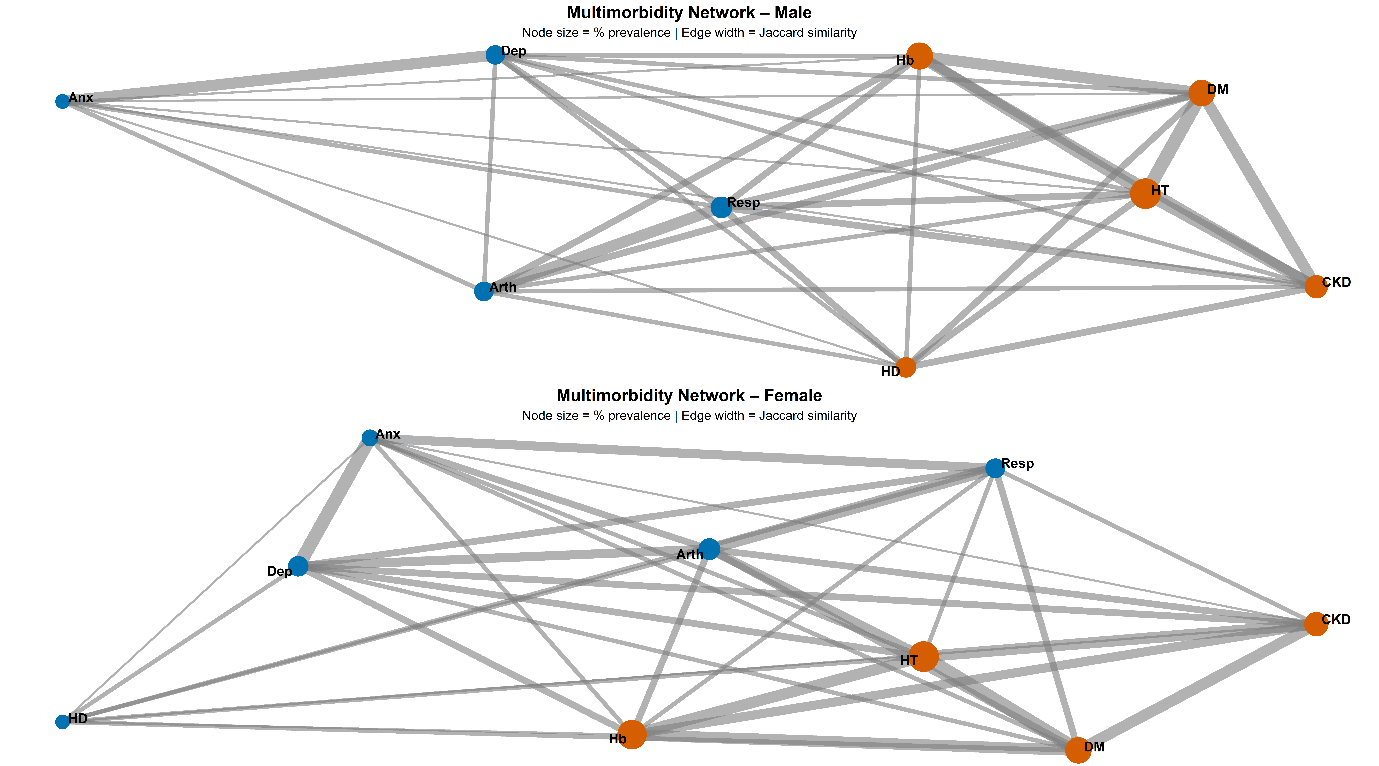


**Figure S1B.** Jaccard-Weighted Multimorbidity Network Analysis by Age Groups


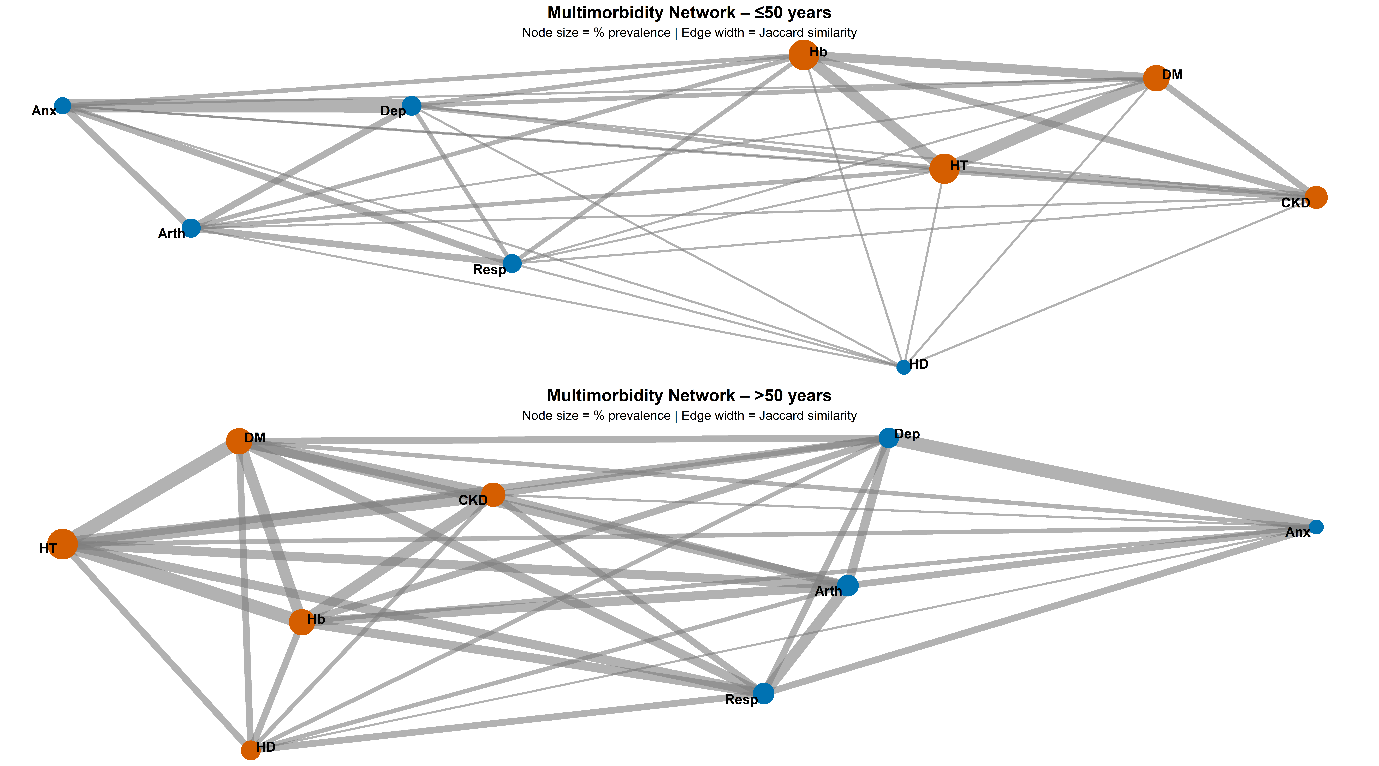


While co-occurrence reflects absolute burden, the Jaccard index reflects relative association strength. The similarity in clustering suggests that the observed multimorbidity patterns are robust and not solely driven by high-prevalence conditions, but represent true underlying disease relationships.

**Figure S2**. Overall Multimorbidity Network Analysis


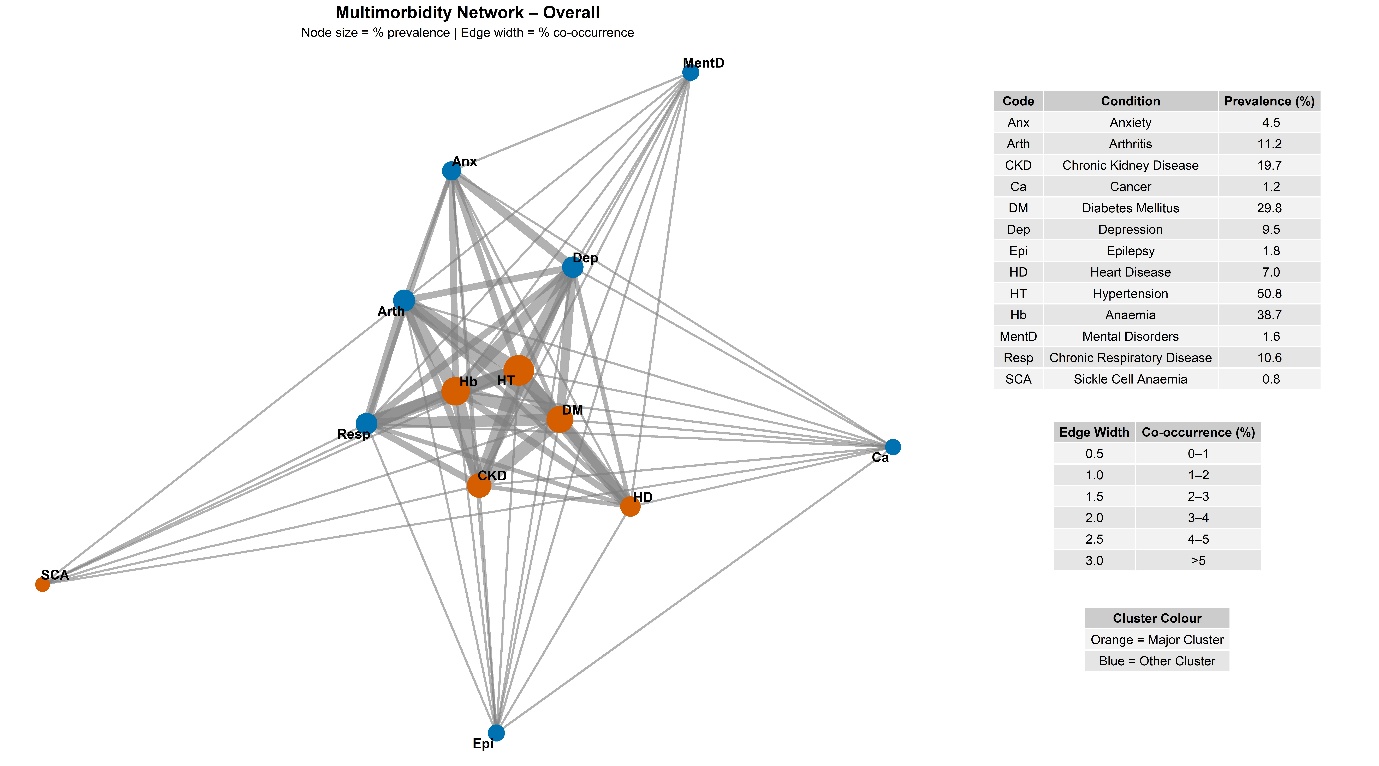

Supplement: Supplementary Figs. S1 and S2 and Tables S1–S14 [file mmc1.docx]
